# Electrocorticographic patterns dominated by low-frequency waves in camphor-induced seizures

**DOI:** 10.1038/s41598-020-75309-w

**Published:** 2020-10-26

**Authors:** Luan Oliveira Ferreira, Rafael Dias de Souza, Fabrício de Araújo Silva, Francisco Fabrizio Moraes Costa, Rômulo Augusto Feio Farias, Akira Otake Hamoy, Vanessa Jóia de Mello, Dielly Catrina Favacho Lopes, Moisés Hamoy

**Affiliations:** 1 Laboratory of Experimental Neuropathology, Federal University of Pará, Belém, 66073-000 Brazil; 2 Laboratory of Pharmacology and Toxicology of Natural Products, Federal University of Pará, Belém, 66077-830 Brazil

**Keywords:** Epilepsy, Neuroscience, Diseases of the nervous system

## Abstract

Camphor is an aromatic terpene compound found in the essential oils of many plants, which has been used for centuries as a herbal medicine, especially in children. However, many studies have shown that camphor may have major side effects, including neurological manifestation, such as seizures. In the present study, we investigated the electrocorticographic patterns of seizures induced by camphor in male adult Wistar rats. Each rat received 400 mg/kg (i.p.) of camphor prior to monitoring by electrocorticography. The application of camphor resulted a rapid evolution to seizure and marked changes in the electrocorticographic readings, which presented characteristics of epileptiform activity, with an increase in the total power wave. The decomposition of the cerebral waves revealed an increase in the delta and theta waves. The analysis of the camphor traces revealed severe ictal activity marked by an increase in the polyspike wave. Our data thus indicate that camphor may cause seizures, leading to tonic–clonic seizures. Clearly, further studies are necessary to better elucidate the mechanisms through which camphor acts on the brain, and to propose potential treatments with anticonvulsant drugs that are effective for the control of the seizures.

## Introduction

Camphor is an aromatic terpene compound present in the essential oil of *Cinnamomum camphora* and a number of other plants, which is either white in color or transparent, and has a characteristic aroma. Camphor has been used for centuries as a herbal medicine, nasal decongestant, analgesic, antiseptic, and insecticide, and also in religious rituals. It is used in the form of an essential oil, oil, spray or isolated compound, and is absorbed rapidly through the skin or digestive tract, or by inhalation^1,2^.

Despite this long history of use, camphor causes a number of side effects, including nausea, vomiting, respiratory impairment, abortive effect, liver and kidney damage, and, in particular, seizures^1–4^. It has been estimated that 75% of the population of the continent of Asia use camphor or camphor-containing compounds in some way^5^. In its most recent annual reports, the American Association of Poison Control Center’s National Poison Data System (NPDS) recorded, on average, more than 13,000 cases of poisoning by camphor or combinations of camphor-containing compounds^6,7^. In fact, a dose of between 50 and 500 mg/kg of camphor may provoke lethal symptoms in children, and a dose of 1 g/kg may have a similar result in adults^1,2,8^. These effects begin 5–20 min after the administration of the camphor, including symptoms such as abdominal pain, vomiting, delirium, anxiety, hallucinations, and tonic–clonic seizures. Death from respiratory failure or seizure may also occur^2^.

It is interesting to note that most cases of accidental poisoning by camphor occur in children, which may evolve to epilepsy with severe neurological sequelae^1–4,8,9^. The mechanisms through which camphor affects brain activity are poorly known, and its relationship with diseases of the central nervous system remains unclear. The diversity of behavioral, psychiatric, and motor effects provoked by camphor poisoning reinforce the need for more systematic data on the action of this compound in the brain, the modifications it provokes, and the similarities with other neuropathologies^2^. The present study describes the electrocorticographic changes in a murine model of camphor-induced seizure, with the objective of identifying the cerebral focus and contributing to the development of effective therapeutic strategies.

## Results

### Behavior and brain wave patterns in camphor-induced seizure

A rapid evolution to seizure was observed in the animals injected with camphor (CPR). The first stage of the seizure (stage 1) included the raising the vibrissae and immobility, and occurred 202.4 ± 13.8 s after the injection of camphor, followed by head jerking (stage 2), at 248.4 ± 13.3 s. Stage 3, which involved spasms of the forelimbs, began at 281.9 ± 26.5 s, while the final stage was characterized by clonic seizure without any transient loss of the posturing reflex, followed by focal seizure that evoked secondary generalization, starting at 566.0 ± 122.7 s. This final stage lasted 24.56 ± 9.4 min, on average.

**Figure 1:**
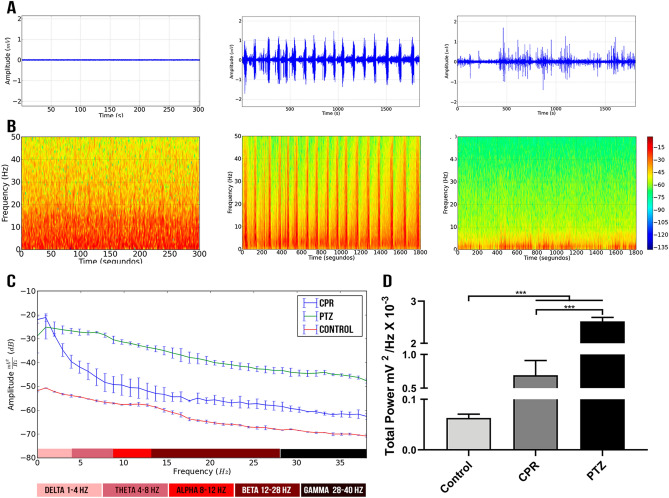
Electrocorticographic recordings and linear frequency distributions in the control group and animals treated with PTZ and CPR. **(A)** 5-min ECoG trace of the control group (left), and 30-min traces for the PTZ (center) and CPR (right) groups. **(B)** Spectrogram of the frequencies of the control (left), PTZ (center) and CPR (right) groups. **(C)** The spectral power distribution of the delta, theta, alpha, beta, and gamma cerebral waves in the three study groups. **(D)** Distribution of the linear frequency of up to 40 Hz in the three groups. The data are expressed as the means ± SD (n = 9 animals per group; *** *p* < 0.0001). (PTZ: pentylenetetrazole; CPR: camphor). **(A–C)** were created using software Python version 2.7, developed by and maintained here: Python Software Foundation, https://www.python.org/downloads/windows/.

The electrocorticographic (ECoG) control presented a regular trace with amplitude of 0.04 mV (Fig. 1A, left) with the majority of the spectral power below 10 Hz, which is normal (Fig. 1B, left). The ECoG of the pentylenetetrazol (PTZ) group (Fig. 1A, center) was characterized by oscillations with peaks of amplitude, typical of a seizure. A cyclical pattern of generalized polyspike-wave discharges (GPWD) can also be noted during wakefulness, with a trace amplitude of 1 mV and the spectral amplitude density increasing to higher frequencies (Fig. 1B, center).

In the case of the camphor, there was a latency of 566.0 ± 122.7 s to the onset of the seizure induced by the substance. The ECoG traces are compatible with a seizure, characterized by repeated and irregular spike-waves and polyspike-wave discharges (Fig. 1A, right), with mean wave amplitude of 1 mV. The ECoG trace of the camphor-induced seizure also presented a prevalence of low-frequency waves, which was also observed in the frequency strength distribution of up to 50 Hz (Fig. 1B, right).

The decomposition of the spectral power distribution revealed greater amplitude of low frequency waves in the camphor group than in the control group. However, the PTZ group presented the greatest oscillations in amplitude in all the waves (see Fig. 1C). Significant variation was found among the groups (F_(2, 22)_ = 625.9; *p* < 0.0001; Fig. 1D) in the distribution of the linear frequencies of up to 40 Hz. A total wave power of 0.06318 ± 0.007428 mV^2^/Hz × 10^−3^ was recorded in the animals of the control group. The total wave power of the camphor group was significantly higher than the control (CPR: 0.6917 ± 0.2197 mV^2^/Hz × 10^−3^; *p* < 0.0001), but lower than the PTZ group (2.522 ± 0.09312 mV^2^/Hz × 10^−3^; *p* < 0.0001), which presented the highest total wave power (*p* < 0.0001 vs control group).

**Figure 2:**
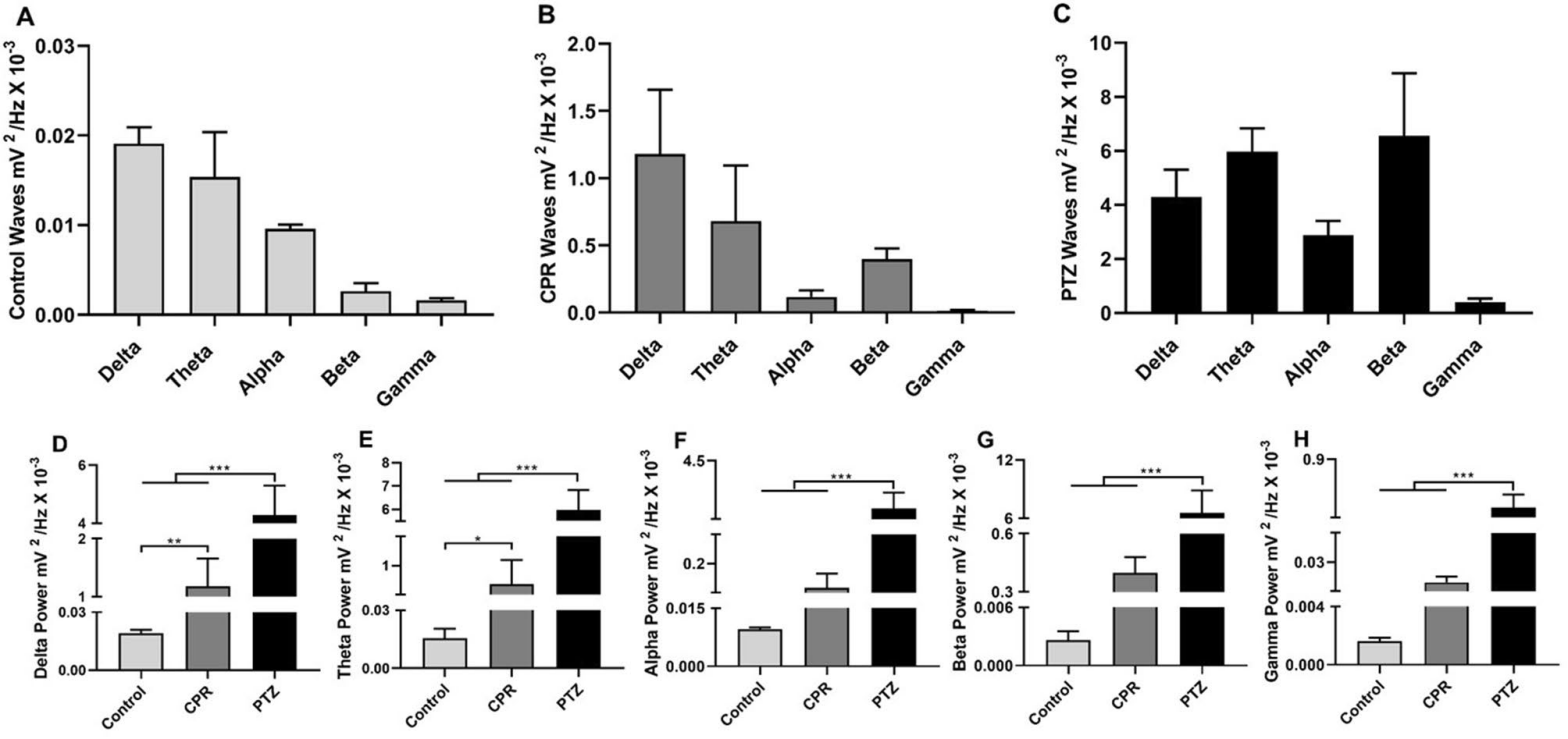
Decomposition of the brain waves in the control animals and during seizures induced by pentylenetetrazole or camphor. **(A)** Decomposition of the brain waves in the control animals. **(B)** Decomposition of the brain waves during seizure induced by pentylenetetrazole. **(C)** Decomposition of the brain waves during seizure induced by camphor. **(D)** Linear frequency distribution of the delta waves. **(E)** Linear frequency distribution of the theta waves. **(F)** Linear frequency distribution of the alpha waves. **(G)** Linear frequency distribution of the beta waves. **(H)** Linear frequency distribution of the gamma waves. The data are expressed as the mean ± SD (n = 9 animals per group; *p = 0.05, **p = 0.01 and ***p < 0.0001) (*PTZ* pentylenetetrazole;* CPR* camphor).

The cerebral waves were also decomposed in the three groups (Fig. 2A–C). Significant variation was found among groups (F_(2, 24)_ = 105.7, *p* < 0.0001) in the configuration of the delta waves, with the PTZ group (4.29 ± 1.01 mV^2^/Hz × 10^–3^) having a 3.6-fold greater delta power than the camphor group (1.18 ± 0.48 mV^2^/Hz × 10^–3^; *p* < 0.0001), with both being significantly higher than the control group (0.02 ± 0.002 mV^2^/Hz × 10^–3^; p < 0.0001 vs PTZ and *p* = 0.0023 *vs* CPR; Fig. 2D). Significant variation was also found among groups in the theta waves (F_(2, 24)_ = 310.5, *p* < 0.0001). The PTZ and CPR groups both presented increased oscillations in the delta waves in comparison with the control group (control: 0.015 ± 0.005 mV^2^/Hz × 10^–3^ *vs* PTZ: 5.97 ± 0.87 mV^2^/Hz × 10^–3^, *p* < 0.0001; and vs CPR: 0.68 ± 0.41 mV^2^/Hz × 10^–3^; *p* = 0.0454). There was also an 8.8-fold increase in the delta wave oscillations in the PTZ group compared with the CPR group (*p* < 0.0001; Fig. 2E).

In contrast with the pattern recorded for the delta and theta waves, in the case of the waves of above 8 Hz, such as the alpha, beta and gamma waves, while significant variation was found among treatments overall, there was no significant difference between the control and camphor groups. Surprisingly, in fact, these waves were responsible for the greatest differences observed between the PTZ and CPR groups. In the case of the alpha waves, despite the significant overall variation (F_(2, 24)_ = 248.9, *p* < 0.0001), the camphor group was not different from the control group (control: 0.01 ± 0.002 mV^2^/Hz × 10^–3^ vs CPR: 0.12 ± 0.05 mV^2^/Hz × 10^–3^,* p* = 0.7441), although the alpha power of the PTZ was more than 23-fold greater than that recorded in the CPR group (PTZ: 2.875 ± 0.53 mV^2^/Hz × 10^–3^; *p* < 0.0001 for both groups; Fig. 2F). A similar pattern was observed in the case of the beta waves, with significant variation among groups overall (F_(2, 24)_ = 68.45, *p* < 0.0001), but not between the control and camphor groups (control: 0.003 ± 0.0009 mV^2^/Hz × 10^–3^ and CPR: 0.40 ± 0.08 mV^2^/Hz × 10^–3^; *p* = 0.8063). By contrast, the PTZ group presented a 16.4-fold increase in beta power in comparison with the CPR group (PTZ: 6.56 ± 2.31 mV^2^/Hz × 10^–3^; *p* < 0.0001 for both comparisons; Fig. 2G). In the case of the gamma waves (F_(2, 24)_ = 79.49, *p* < 0.0001), the PTZ presented a 25-fold increase in the wave power in comparison with the camphor group (PTZ: 0.40 ± 0.13 mV^2^/Hz × 10^–3^ *vs* CPR: 0.016 ± 0.004 mV^2^/Hz × 10^–3^; *p* < 0.0001; Fig. 2H). Similarly, a significant difference was found only between the control and the PTZ group (control: 0.002 ± 0.0003 mV^2^/Hz × 10^–3^ *vs* PTZ group, *p* < 0.0001) and not in comparison with the camphor group (*p* = 0.9155). Clearly, then, our findings indicate that camphor-induced seizures alter only low-frequencies waves.

### Characteristics and decomposition of the camphor ECoG

**Figure 3:**
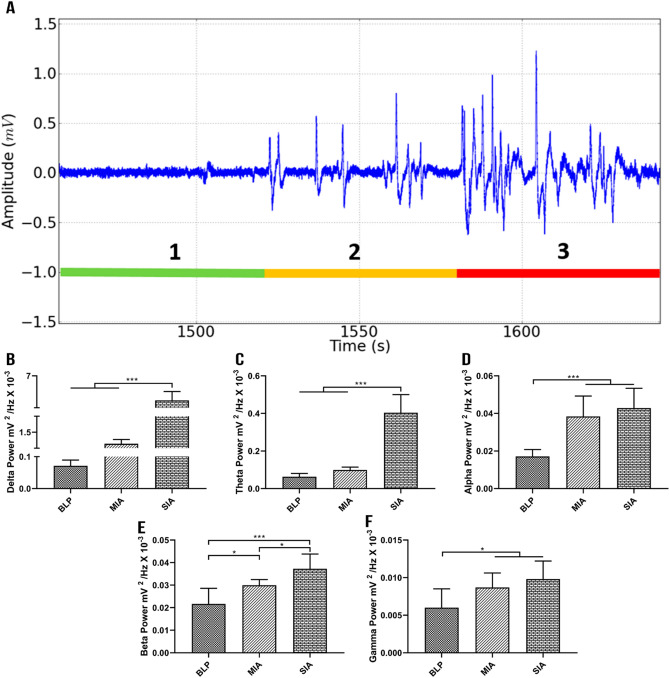
Decomposition of the brain waves in the control animals and during seizures induced by pentylenetetrazole or camphor. **(A)** 200 ms fragment of the camphor trace (1: baseline period (BLP); 2: mild ictal activity (MIA); 3: severe ictal activity (SIA)). **(B)** Linear frequency distribution of the delta waves during the camphor-induced seizure. **(C)** Linear frequency distribution of the theta waves during the camphor-induced seizure. **(D)** Linear frequency distribution of the alpha waves during the camphor-induced seizure. **(E)** Linear frequency distribution of the beta waves during the camphor-induced seizure. **(F) **Linear frequency distribution of the gamma waves during the camphor-induced seizure. Data are expressed as the mean ± SD (n = 9 animals per group; *p < 0.05 and ***p < 0.0001). **(A)** was created using software software Python version 2.7, developed by and maintained here: Python Software Foundation, https://www.python.org/downloads/windows/.

To better understanding the pattern of the camphor-induced seizure, we cut extracted a 200 ms fragment of the ECoG (Fig. 3A) and evaluated the frequency band activity during the Baseline Period (BLP) and two stages of ictal activity (mild and severe). Spike-wave (SWD) discharges are clearly visible in the Mild Ictal Activity (MIA), which become more intense in the Severe Ictal Activity (SIA), with intense spike-waves (SW) and polyspike and sharp wave discharges (PSWD).

Significant variation was found among phases in the delta waves (F_(2, 24)_ = 87.17, *p* < 0.0001), although the difference was not significant between BLP and MIA (BLP: 0.07 ± 0.02 mV^2^/Hz × 10^–3^ and MIA: 0.78 ± 0.26 mV^2^/Hz × 10^–3^; *p* = 0.0787). However, the delta oscillations were significantly higher in the SIA in comparison with the other phases (SIA: 3.93 ± 1.11 mV^2^/Hz × 10^–3^; *p* < 0.001 for both comparisons; Fig. 3B). In the case of the theta waves, while significant variation was also found among groups (F_(2, 24)_ = 95.76, *p* < 0.0001), significant differences were found only between the SIA and the other two phases (BLP: 0.06 ± 0.02 mV^2^/Hz × 10^–3^; MIA: 0.10 ± 0.015 mV^2^/Hz × 10^–3^; SIA: 0.40 ± 0.10 mV^2^/Hz × 10^–3^; *p* < 0.0001 for BLP *vs* SIA and MIA *vs* SIA; Fig. 3C).

In the case of the alpha waves (F_(2, 24)_ = 21.19, *p* < 0.0001), significant differences were found between BLP (BLP: 0.02 ± 0.004 mV^2^/Hz × 10^–3^) and ictal activity (MIA: 0.038 ± 0.01 mV^2^/Hz × 10^–3^; SIA: 0.042 ± 0.01 mV^2^/Hz × 10^–3^; *p* < 0.0001 *vs* both MIA and SIA), but not between the SIA and MIA (*p* = 0.5447; Fig. 3D). In contrast with all the other waves, the beta waves varied significantly both overall (F_(2, 24)_ = 16.83, *p* < 0.0001), and in all pairwise comparisons. The lowest amplitude was recorded in the baseline period (0.022 ± 0.006 mV^2^/Hz × 10^–3^) and the highest in the SIA (MIA: 0.030 ± 0.002 mV^2^/Hz × 10^–3^; *p* = 0.0133 vs BLP; SIA: 0.037 ± 0.006 mV^2^/Hz × 10^–3^; *p* < 0.0001 *vs* BLP and *p* = 0.0321 *vs* MIA; Fig. 3E). As for the alpha waves, the gamma waves varied significantly among phases (F_(2, 24)_ = 6.601, *p* = 0.0052), although no differences was found between the ictal activity phases (MIA: 0.009 ± 0.002 mV^2^/Hz × 10^–3^; SIA: 0.01 ± 0.002 mV^2^/Hz × 10^–3^; *p* = 0.5639), although both these periods were significantly higher than the baseline period (BLP: 0.006 ± 0.002 mV^2^/Hz × 10^–3^; *p* = 0.0497 vs MIA and *p* = 0.0047 vs SIA; Fig. 3F).

## Discussion

Herbal medicines and essential oils are popular worldwide, especially in oriental medicine, in China, and other parts of Asia. These substances are used for a diverse range of complaints, including coughs, colds, microbial infections, and pain, and are used by approximately 75% of the population in Asia^5^. However, the potential toxicity of herbal medicines has received little attention, especially when taken in unknown quantities in composite substances^10^. There are, however, many case reports of herbal medicines and essential oils causing severe toxicity or even death, and these cases include camphor, whether applied topically, inhaled or orally^2^.

Camphor is used in a range of pharmaceutical formulations, including ointments, oils, vaporizer solutions, patches, mothballs or in its solid form^2^. The mechanisms of camphor toxicity are poorly known, although Vatanparast and Andalib-Lari^11^ suggested that camphor causes a direct blockade of the K+ channels and an increase in the influx of Ca^2+^ in the neurons, which has serious effects on the brain, such as hallucinations and seizures^2,12^. Camphor may also cause changes in hormones and the reproductive organs^13,14^.

There are many recorded cases of seizures induced by camphor, especially in children^9,12,15–19^. Despite these records, little is known about this phenomenon. The results of the present study show clearly that camphor is capable of inducing tonic–clonic seizures in rats, with effects similar to those described in humans^8,9,12,15,18^. There are many types of seizure, which present different electroencephalographic patterns and behavioral correlates^20,21^. Here, we described the patterns of camphor-induced seizure, and found moderate neuronal hyperexcitability, with a predominance of low-frequency waves (delta and theta), which are related to tonic–clonic seizures^22^. These findings corroborate those of Jalilifar et al.^23^, which showed that motor alterations and behavioral seizure progression are associated with an increase in delta oscillations.

Interestingly, the wave pattern observed in the camphor-induced seizure presented the characteristics of a generalized spike-wave discharge, concentrated in low frequency waves, below 8 Hz (delta and theta). These frequencies may be faster and more irregular at the onset of the seizure and decelerate gradually towards its termination, while intra-discharge irregularities may also occur, as observed in the ECoG traces obtained from the camphor group. The amplitude of these spike-wave complexes typically peaks over the frontal areas and displays an anterior to posterior gradient, frequently fading over the occipital areas^24^. This pattern is very indicative of absence seizures^24^. Given these characteristics and the available data, we propose that camphor causes absence seizures, which evolve to generalized tonic–clonic seizures. Although only a single pair of electrodes was used in the present study, which may result in lower spatial accuracy than other systems, Johnstone et al^25^ and Hemington and Reynolds^26^ confirmed the validity of this approach for EEG records and diagnostics.

**Figure 4:**
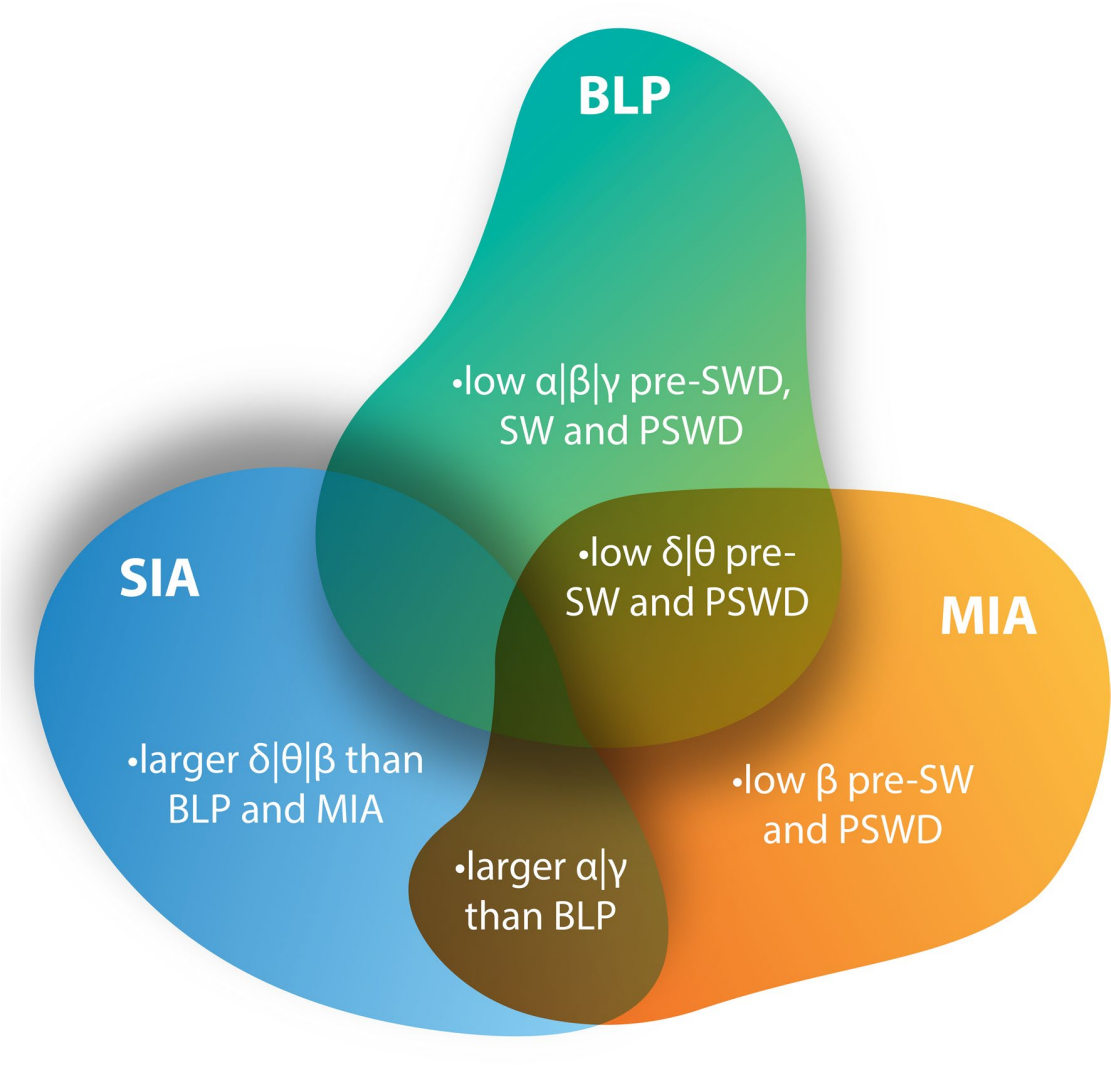
Common features and the key differences in the epileptiform events of camphor-induced seizures. After the baseline, both epileptiform events present the greatest spike voltage in the low frequency waves (delta and theta) and in particular, in the high frequency waves (alpha, beta and gamma), as well as significant increases in β‐spectral density from the baseline levels. Baseline period (BLP); Mild Ictal Activity (MIA) and Severe Ictal Activity (SIA). Spike-wave discharges (SWD). Spike-wave (SW). Polyspike and sharp wave discharges (PSWD). The figure was created using software Adobe Photoshop CS6 developed by and maintained here: https://www.adobe.com/br/products/photoshop.html#.

In the decomposition of the camphor wave profile (Fig. 3), the delta waves of the baseline and mild ictal activity periods were similar, with a significant increase only being observed in the period of severe ictal activity (Fig. 4). A similar pattern was observed in the case of the theta waves. This supports the hypothesis that the camphor-induced seizure was characterized by low frequency waves, with symptomatic manifestation, as described Jalilifar et al.^27^. The wave profile was also marked by the beta waves, which varied significantly between the three periods (Figs. 3E and 4). The available data do in fact highlight the role of the beta waves in generalized seizures, which indicates that this wave can be used to define the transition between the different morphological states, that is, the baseline (Fig. 3A1), mild ictal activity (Fig. 3A-2, spike-wave), and severe icital activity (Fig. 3A-3, polyspike-wave).

Overall, then, camphor provoked convulsive symptoms, marked by altered behavior and electrocorticographic activity. Changes were also observed in total power and low frequency waves (delta and theta). Our findings show that camphor may cause absence seizures, which may lead to tonic–clonic seizures. This emphasizes the need for further research to better elucidate the mechanisms through which camphor acts on the brain, including whether there is glial cell activity, neuronal death or long-term functional impairment. The preset study nevertheless provides important insights for the development of clinical protocols for the administration of anticonvulsant drugs for seizure control.

## Methods

### Study animals

Twenty-seven adult males Wistar rats (250 ± 30 g) were obtained from the Central Animal Facility of the Federal University of Pará in Belém, Brazil. The animals were housed at a controlled temperature, of 23 ± 2 ºC and 12/12 h light–dark cycle, with food and water available ad libitum. All experimental procedures were conducted in accordance with the principles of laboratory animal care^28^ and the guidelines of the Brazilian National Council for the Control of Animal Experimentation (CONCEA), with the approval of the Ethics Committee on Experiments in Animals of the Federal University of Pará (CEUA no. 8381060818). All necessary procedures were employed to prevent animal suffering and distress.

### Drugs

The anesthetic ketamine was purchased from König (Santana de Parnaíba, SP, Brazil), while xylazine was obtained from Vallée (Montes Claros, MG, Brazil). The convulsive agent pentylenetetrazol was purchased from Sigma-Aldrich (St Louis, USA) and the camphor from Êxodo Científica (Sumaré-São Paulo).

### Experimental design

The rats were divided randomly (*n* = 9 animals per group) into: (a) vehicle-treated group (control); (b) pentylenetetrazole (PTZ) group [positive control for the induction of seizures, 60 mg/kg, intraperitoneally (i.p.)], and (c) camphor (CPR, 400 mg/kg, i.p.)^29^. The dosage was established by linear regression, based on the appearance of clonic seizures without the loss of the posture reflex. Immediately after the administration of the respective substances, the electrocorticographic (ECoG) recordings were initiated and the seizure-related behavior of the camphor group was monitored.

### Description of the seizure-related behavior

Seizure-related behavior was observed following the injection of the camphor, when the animals were maintained in a standard white cage (48 cm × 38 cm × 21 cm). Latency to the seizure was recorded and behavioral modifications were classified in four clinically-identifiable stages, as suggested by Hamoy et al.^30^: (1) raising the vibrissae and immobility, (2) head jerk, (3) forelimb spasms, and (4) clonic seizure without any loss of the posturing reflex.

### Electrocorticographic recordings and data analyses

The ECoG recordings and offline data analyses followed the procedures described by Estumano et al.^31^. The animals were first anesthetized with ketamine hydrochloride (80 mg/kg, i.p.) and xylazine hydrochloride (10 mg/kg, i.p), and once the corneal reflex had been abolished, they were placed in a stereotaxic apparatus and stainless steel electrodes (1.0 mm diameter tip exposure) were placed on the duramater at the coordinates of the bregma—0.96 mm and ± 1.0 mm lateral. Five days after surgery, the ECoGs were obtained using a digital data acquisition system and the offline analyses were conducted. The analyses were run at a frequency of up to 50 Hz, and split into bands as in Estumano et al.^31^, that is, delta (1–4 Hz), theta (4–8 Hz), alpha (8–12 Hz), beta (12–28 Hz), and gamma (28–40 Hz), for interpretation of the wave dynamics during the development of the seizure.

The recordings followed a predefined protocol: the animals were immobilized carefully for 10 min for habituation, to avoid interference in the records. The basal ECoG activity was then recorded for 30 min, for use as the control in the ECoG analyses. The PTZ (positive control) or camphor was then administered, and the ECoG activity was recorded for a further 30 min. The animals were then euthanized to avoid further distress.

### Statistical analyses

The normality of the data was verified using the Kolmogorov–Smirnov test, and the homogeneity of the variances was confirmed by the Levene statistic. As the residuals were distributed normally and the variances were homogeneous, comparisons of the mean amplitudes of the treatment and control values were based on a one-way ANOVA followed by Tukey’s test for multiple comparisons. The data are presented as the mean ± standard deviation (SD), and the F and p values are included, when pertinent. A p < 0.05 significance level was considered for all analyses. All statistical procedures were un in the GraphPad Prism software, version 8 (Graph-Pad Software Inc., San Diego, CA, USA).
